# Limited public understanding of the risk factors and complications of hypertension

**DOI:** 10.1097/HJH.0000000000004233

**Published:** 2026-01-09

**Authors:** Mingjuan Zeng, Sonali R. Gnanenthiran, David K.E. Chan, Ruth Griffiths, Aletta E. Schutte

**Affiliations:** a The George Institute for Global Health; b School of Population Health; c South West Sydney Clinical Campuses, Discipline of Surgery, School of Clinical Medicine, University of New South Wales; d Cardiology Department, Concord Repatriation General Hospital; e Stats Central, Mark Wainwright Analytical Centre, University of New South Wales, Sydney, NSW; f Department of Health, Disability and Ageing, Phillip, ACT, Australia

**Keywords:** Australia, blood pressure, cardiovascular complications, education, hypertension, knowledge, risk factors

## Abstract

**Objective:**

We evaluated Australian adults’ knowledge on the risk factors and complications of hypertension to identify areas of poor understanding.

**Methods:**

In 2024, an adapted validated hypertension-knowledge survey was distributed to participants who had used kiosk BP stations in retail stores across metropolitan and remote Australia. Kiosk stations collected measured and self-reported data.

**Results:**

A total of 826 participants (51% male; mean age 52 ± 16 years; 36% with a history of hypertension) completed the survey. Participants generally demonstrated a good understanding of hypertension risk factors, with 90% recognizing that being overweight increases risk, and that exercise helps lower BP. Twenty-seven percent were unaware that hypertension is usually asymptomatic; 41% did not know that stress is not its main cause; and 42% were unaware of the importance of medication adherence. Seventy-three percent and 77% were unaware of the increased risk of dementia and kidney disease, respectively.

People with a history of hypertension (vs. no hypertension; *P* = 0.01) and those in outer regional areas (vs. major cities, inner regional, or remote; *P* = 0.011) had better knowledge. Individuals who were unemployed or had lower educational backgrounds (both *P* < 0.001) had poorer knowledge. Kiosk BP readings and the use of antihypertensive medications were not associated with knowledge scores.

**Conclusions:**

Public misconceptions regarding hypertension persist. With three in four participants unaware that hypertension increases the risk of dementia and kidney disease, and with limited understanding of the importance of medication adherence, educational efforts are essential, particularly among people who are unemployed or have lower educational backgrounds.

## INTRODUCTION

Hypertension is the leading risk factor for mortality worldwide [1,2]. It contributes to kidney failure, heart failure, coronary artery disease, cardiac arrhythmia, stroke and dementia [3–5]. Hypertension is poorly controlled, both globally and in Australia [6–9]. In Australia, the National Hypertension Taskforce was established to improve blood pressure (BP) control from the current rate of 32–70% by 2030 through prevention, detection and effective treatment [10,11]. Greater public awareness, knowledge and understanding of the risks and complications of hypertension may help achieve this goal.

Among individuals with hypertension, studies in China, Malaysia, Poland and Israel have found that patients with greater knowledge of hypertension also had healthier lifestyles, better awareness, improved medication adherence, and better BP control [12–16]. For those at risk of developing hypertension, a randomized controlled trial in China involving 607 participants demonstrated that a 1-year community programme – including education, regular general practitioner (GP) visits, and lifestyle self-management – significantly reduced the incidence of hypertension, with participants in the intervention group also showing greater hypertension knowledge and monitoring their BP more frequently [17]. However, few studies have examined the relationship between public understanding of hypertension and BP levels. A cross-sectional study in Italy involving 2731 participants found that individuals with lower hypertension knowledge scores had significantly higher BP values compared to those with greater knowledge [18].

The level of hypertension knowledge in Australia remains unclear. A study more than two-decades ago assessed hypertension knowledge in a small sample of 55 people with hypertension [19]. Most research on public knowledge has been conducted in countries with differing levels of health literacy, and it is thus unclear whether this can be translated to the Australian setting [18,20–24]. We therefore aimed to evaluate hypertension knowledge in Australian adults across metropolitan, regional and rural areas. We also sought to identify factors that best predict knowledge scores.

## METHODS

This study was embedded within the Shop-to-Stop Hypertension clinical trial performed in New South Wales, Australia, which investigates whether text message-based nudges influence health-seeking behaviours in people detected with raised BP at health kiosks [25]. The trial included 30 hardware chain stores which were selected based on their location to represent remote, rural, regional and metropolitan populations. Each store was equipped with a SiSU Health Station (SiSU Health Group, Australia) in an area that was easily accessible. Any person could voluntarily perform their own health screening at a Health Station without the assistance of another person. When a person accesses a Health Station for the first time, a user profile (with an E-Mail address and password) is created on the Health Station, and the results of the screening are automatically emailed to the person afterwards. The trial and this sub-study were approved by the University of NSW (UNSW) Human Research Ethics Committee (HREC) (Reference Number: iRECS5031). Participants gave consent to participate in the study.

### Recruitment

All participants aged 18 and over who used a SiSU Health station between November 2023 and November 2024 as part of the larger trial were eligible to receive an E-Mail invitation to participate in a survey on their understanding of hypertension. Emails were sent in three batches to a total of 25 162 participants: the first to 500 participants, the second to 778 participants and the final batch to 23 884 participants. A single E-Mail reminder was sent to those who did not respond. Participants had the opportunity to enter a raffle to win one of 15 $100 gift vouchers.

### Data collection

The SiSU Health stations collected both machine-measured and self-reported data. The machine-measured data included measured height and weight (to calculate body mass index (BMI)), BP and heart rate. Self-reported data from the SiSU Health kiosk questionnaire included age, gender, postcode of their primary residency and health information, such as the use of antihypertensive medications, diabetes status and smoking status. A question on hypertension literacy assessed participants’ awareness of various complications of hypertension. The questionnaire also included an optional diabetes risk assessment for participants without a current diabetes diagnosis. This assessment inquired about participants’ family history of diabetes, country of birth, daily fruit and vegetable intake, and exercise duration (SiSU Health questionnaire in Table S1, Supplemental Digital Content).

The survey was developed using SurveyMonkey and distributed to participants via E-Mail (Table S2, Supplemental Digital Content). The survey gathered participant demographics, including educational levels, employment status, and family history of cardiovascular-related diseases. It also included a hypertension knowledge survey, which covered risk factors, diagnosis, lifestyle, medication management, and complications. The knowledge survey was adapted and combined from two validated hypertension knowledge scales from the United States [26,27], and included two parts: 13 true/false questions, and five single-choice questions and one multiple-choice question.

### Data analysis

Data were analysed using R, version 4.4.1 [28]. Descriptive statistics were used to summarize continuous and categorical data. Based on normality testing from the Shapiro–Wilk test, continuous data are presented as mean and standard deviation (SD) or median and interquartile range (IQR). Categorical data were reported as numbers and percentages. Each participant's knowledge score was calculated using the method listed in Table S2, Supplemental Digital Content. The association between knowledge scores and BP levels was evaluated using Spearman's rank correlation coefficient (*ρ*) test. To identify predictors for knowledge scores, we used multiple linear regression with the individual knowledge score as the dependent variable. The predictor variables included: age group, gender, BP readings, BMI, history of hypertension, current antihypertensive medication use, smoking status, diabetes status, family history of cardiovascular-related diseases (hypertension, heart disease and stroke), education level, employment status, geographical remoteness, socio-economic status, when the participant last saw a GP, BP measurement recency, and number of kiosk visits. The predictors in the final model were selected based on the adaptive least absolute shrinkage and selection operator (Adaptive LASSO), which ensures consistent predictor selection [29]. A subgroup analysis was performed on participants with a history of hypertension. Participants who selected “Prefer to not say” for any predictor variable were excluded from the final model and the subgroup analysis as the numbers were small. However, a sensitivity analysis was conducted using the final model for the total study population, incorporating these participants to evaluate their influence on the regression results. To assess whether lifestyle factors, including daily fruit and vegetable intake, and exercise duration influenced knowledge score, we conducted a Wilcoxon rank-sum test. This was limited to participants without a diabetes diagnosis, as lifestyle factors data were not collected for those with diabetes. Additionally, we reapplied the adaptive LASSO procedure for this subgroup to determine whether these lifestyle factors were significant predictors of knowledge scores.

## RESULTS

### Sociodemographic characteristics of respondents

A total of 826 participants (3.5% response rate; 50.6% male; mean age 52 ± 16 years; mean BP 122/74 ± 19/11 mmHg) completed the survey (Table 1). Of those, 36.3% had a history of hypertension, and 23% were using antihypertensive medications. Eighteen percent had a high BP reading as measured at the kiosk, defined as BP exceeding 140 and/or 90 mmHg. Among those with a history of hypertension, 35.6% had a high BP reading. Most participants lived in major cities (45.4%) or inner regional areas (40.7%). While a greater proportion (19.5%) of participants were from the highest socio-economic decile, there was an overall good spread across the socio-economic classes. Most participants obtained health information from their healthcare professional (93%), followed by the internet (61%). Table S3, Supplemental Digital Content compares the characteristics of survey respondents and nonrespondents. Respondents included a higher proportion of females, were older, and were more likely to have higher systolic BP readings and to be taking antihypertensive medications (all *P* < 0.001). Respondents and nonrespondents were similar in terms of geographic remoteness, BMI, and diastolic BP readings.

**TABLE 1:** Sociodemographic characteristics of participants

Variable (*N* = 826)	*n*/mean	%/SD
Age in years	52	±15.8
Age group		
18–34 years	132	16.0
35–44 years	159	19.2
45–54 years	143	17.3
55–64 years	180	21.8
65–74 years	157	19.0
75 years and above	55	6.7
Female/male	408/418	49.4/50.6
Body composition		
Body mass index, kg/m^2^ (*n* = 782)	28.1	5.47
Blood pressure		
Systolic BP, mmHg (*n* = 792)	122	19
Diastolic BP, mmHg (*n* = 792)	74	11
BP > 140/90 mmHg (*n* = 792)	145	18.3
History of hypertension	300	36.3
BP>140/90 mmHg (*n* = 289)	103	35.6
Using antihypertensive medications	199	24.1
Diabetes (*n* = 788)	56	7.1
Daily smoker (*n* = 788)	46	5.8
Family history		
Hypertension	407	49.3
Heart disease	269	32.6
Stroke	169	20.5
BP measured last 12 months (*n* = 817)	526	64.4
Last saw general practitioner (*n* = 816)		
1 month ago	426	52.2
6 months ago	243	29.8
6–12 months ago	41	5.0
More than 12 months ago	52	6.4
Cannot remember	54	6.6
Eating vegetable and fruit daily (*n* = 678)	576	85.0
Physically active (>2.5 h/week) (*n* = 678)	525	77.4
Highest level of education		
Year 10 certificate or equivalent or less	146	17.7
Year 12 or equivalent	80	9.7
Vocational qualification	205	24.8
University degree	381	46.1
Prefer not to say	14	1.7
Employment status		
Employed full-time	323	39.1
Employed part-time	176	21.3
Unemployed	43	5.2
Retired	218	26.4
Not working/permanently unable to work	43	5.3
Prefer not to say	16	1.9
Geographical remoteness^a^ (*n* = 821)		
Major cities	375	45.7
Inner regional	334	40.7
Outer regional	103	12.5
Remote and very remote communities	9	1.1
Socio-economic status^b^ (*n* = 821)		
1	56	6.8
2	70	8.5
3	55	6.7
4	133	16.2
5	121	14.7
6	71	8.6
7	47	5.7
8	54	6.6
9	54	6.6
10	160	19.5
Country of birth (*n* = 678)		
Australia	495	73.0
Southern Europe	14	2.1
Asia	66	9.7
Middle East	7	1.0
Other	96	14.2
Aboriginal and Torres Strait Islander (*n* = 678)	20	2.9
Obtain health information from:		
Healthcare professionals	764	92.5
Internet	505	61.1
Social media	90	10.9
Family and friends	218	26.4
Television	74	9.0
Print media	81	9.8
Other	41	5.4

a Postcode was used to classify geographical remoteness according to the Australian Statistical Geography Standard – Remoteness Areas (ASGS-RA) framework [30].

b Postcode was used to classify socio-economic status according to the Index of Relative Socio-Economic Advantage and Disadvantage, which summarizes individual and household economic and social information to create a place-based indicator of relative advantage and disadvantage. A decile score of 1 indicates the most disadvantaged (lowest socio-economic status), while a score of 10 indicates the most advantaged (highest socio-economic status) [31].

### Knowledge of risk factors, diagnosis, management and complications

Over 90% of participants correctly identified that hypertension can affect both the old and young, and that being overweight is a risk factor for hypertension, while exercise helps lower BP. Forty-one percent failed to understand that stress is not the main cause of high BP. In terms of diagnosis, 82% of participants understood that high BP readings are required to diagnose high BP, with 65% understanding the correct diagnostic threshold. Furthermore, 27% of participants were unaware that high BP may not cause symptoms.

Regarding the management of hypertension, 45% failed to identify a diet that helps lower BP, and 42% did not understand the importance of taking antihypertensive medications regularly even on the days when they feel well. Regarding knowledge about the complications of hypertension: 23% failed to associate hypertension with heart attacks, 55% did not link hypertension with stroke, 73% were unaware of its connection to dementia risk, and 77% did not recognize the increased risk of kidney disease. However, when stroke was presented as an option later in the survey, 92% of participants were able to identify it. Correct response rates are presented in Table 2.

**TABLE 2:**
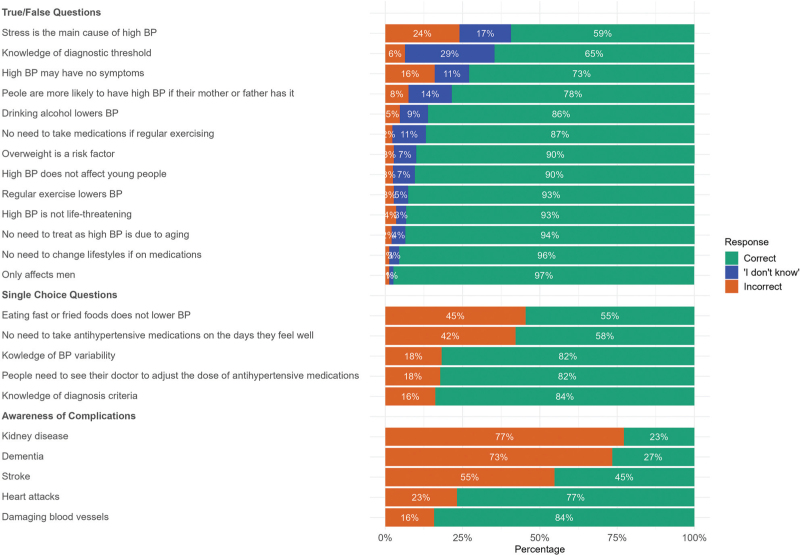
Participants’ knowledge based on different types of questions

### Association of participant knowledge scores and BP values

There was no statistically significant correlation between participant knowledge scores and either systolic or diastolic BP in the total study population, in participants with a history of hypertension or in participants using antihypertensive medications. A Spearman's rank correlation test showed a very weak negative association between systolic BP and knowledge score (*ρ* = −0.042), and a very weak positive association between diastolic BP and knowledge score (ρ= 0.038).

### Individuals’ knowledge levels based on different predictors

Variables including BP readings, BMI, current antihypertensive medication use, diabetes status, socio-economic status, recent BP measurement, and number of kiosk visits, were found not to associate with knowledge scores and were excluded from the final multiple linear regression model. Predictors included age group, gender, history of hypertension, smoking status, family history of cardiovascular-related diseases (hypertension, heart disease and stroke), education level, employment status, geographical remoteness, and when they last visited a GP. The final model was statistically significant (*F*(27, 714) = 5.89, *P* < 0.001) explaining 15.1% of the variance (*R*^2^ = 0.182, adjusted *R*^2^ = 0.151).

**FIGURE 1:**
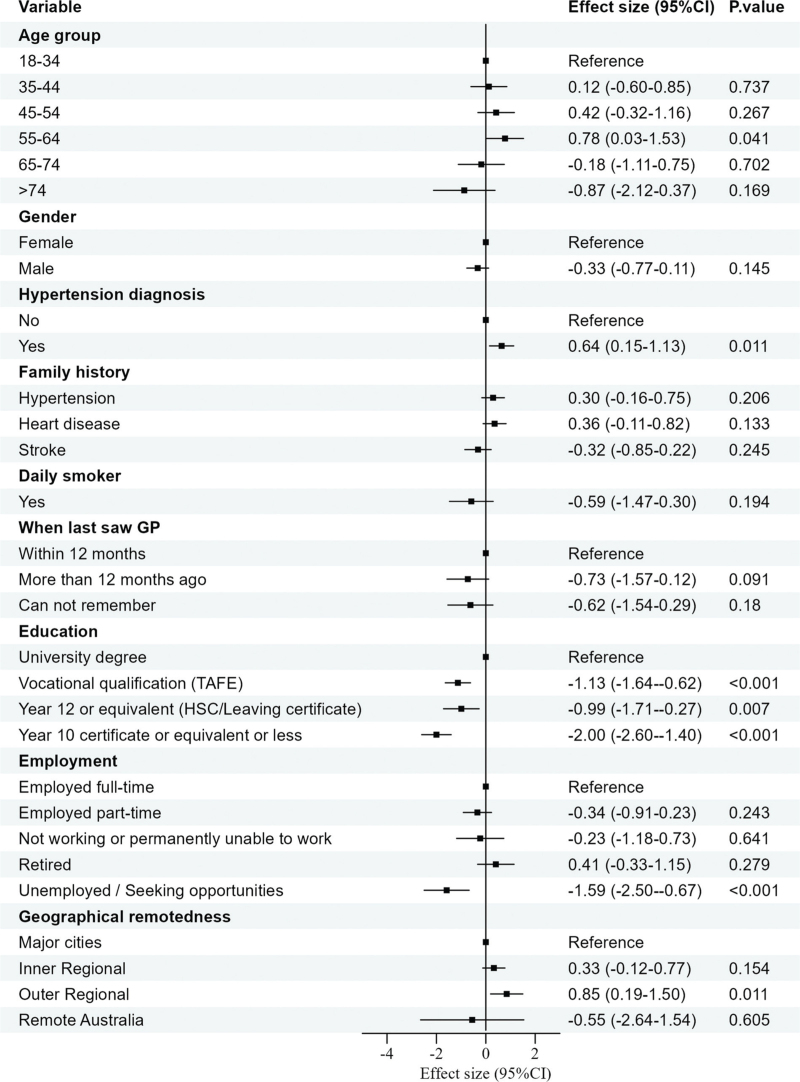
Effect sizes for predictors of hypertension knowledge scores.

As shown in Fig. 1, compared to people aged 18–34 years, those aged 55–64 years had greater knowledge (*β* = 0.78, 95% CI = 0.03–1.53, *P* = 0.041), with no differences observed in other age groups. Individuals with a previous hypertension diagnosis scored better (*β* = 0.64, 95% CI = 0.15–1.13, *P* = 0.010). Education level was associated with participant knowledge scores. Compared to individuals with a university degree, those with a vocational qualification (*β* = −1.06, 95% CI = −1.55 to −0.57, *P* < 0.001), Year 12 education (*β* = 0.93, 95% CI = −1.62 to −0.23, *P* = 0.009), and Year 10 education (*β* = −1.82, 95% CI = −2.42 to −1.27, *P* < 0.001) had significantly lower scores. Regarding employment status, individuals who were unemployed or seeking opportunities had lower knowledge scores compared to those employed full-time (*β* = −1.59, 95% CI = −2.50 to −0.67, *P* < 0.001), while no difference was observed in those who worked part-time, were not working, or were retired. Participants living in outer regional Australia scored higher (*β* = 0.85, 95% CI = 0.19–1.50, *P* = 0.028), with no difference in the other groups when compared to those living in major cities. Gender, family history of cardiovascular-related diseases, and smoking status were included in the model, but were not significant predictors of hypertension knowledge.

Among individuals with a history of hypertension (*n* = 290), age group was not associate with knowledge scores (*P* = 0.65), but education level was associated. Individuals with vocational qualifications (*β* = −1.03, 95% CI = −1.82 to −0.23, *P* = 0.011) or a Year 10 certificate or less (*β* = −1.48, 95% CI = −1.82 to −0.23, *P* < 0.001) demonstrated lower knowledge scores than those with university degrees. There was no difference between those with a university degree and those who had completed secondary school (*P* = 0.62). Compared to those who were employed, unemployment remained a predictor of lower knowledge (*β* = −2.40, 95% CI = −4.01 to −0.78, *P* = 0.004). People living in remote areas of Australia had lower knowledge scores than those in major cities (*β* = −3.03, 95% CI = −5.57 to −0.49, *P* = 0.020). Effect sizes are listed in Figure S1, Supplemental Digital Content.

### Sensitivity analysis

When participants who selected “Prefer not to say” were included in the analysis, the effect sizes and predictors compared to the final model for the total study population remained largely unchanged. One exception is that participants who could not recall when they last saw a GP had lower hypertension knowledge scores compared to those who had seen a GP within 12 months (*β* = −0.92, 95% CI = −1.80 to −0.03, *P* = 0.041) (Figure S2, Supplemental Digital Content).

### Association of participant knowledge scores and lifestyle factors

No differences were observed in hypertension knowledge scores between participants who engaged in regular exercise and those who did not (*P* = 0.54). Similarly, no differences were found between participants who consumed fruits and vegetables daily and those who did not (*P* = 0.10). Also, the adaptive LASSO procedure did not identify these lifestyle factors as predictors of knowledge scores.

## DISCUSSION

This study evaluated the hypertension knowledge of individuals who voluntarily participated in BP screening at kiosks in Australia. We found that although people demonstrated moderate knowledge of many aspects of hypertension, several key elements were poorly grasped. Knowledge of the complications of hypertension was particularly limited, with three out of four participants unaware of its association with kidney disease or dementia. Additionally, many did not know that hypertension is usually asymptomatic, that stress is not its main cause, or the importance of medication adherence. Knowledge gaps were more pronounced among individuals with lower educational attainment and those who were unemployed.

Public awareness of the many complications of hypertension was limited. Before our study, there was little evidence on hypertension knowledge among the general population in Australia, although some research existed among individuals diagnosed with hypertension. A small qualitative study conducted in 2022 on cuffless BP devices in individuals with hypertension found that half did not recognize the link between high BP and heart disease, and three-quarters were unaware of its association with stroke, eye disease and kidney disease [3]. However, a 2003 study, also involving individuals diagnosed with hypertension, reported that 71% were aware of the risk of stroke associated with hypertension [19]. The public's lack of awareness regarding hypertension complications is not unique to Australia. In a 2019 Israeli study of community-dwelling adults, 62% of participants were unable to identify at least two complications of hypertension [23]. Similar to our study, an Italian study found that 72% of people were unaware of the association between hypertension and kidney disease [18], although it did not assess awareness of dementia risk. Consistent with our findings, a systematic review of public awareness regarding cardiovascular risk factors for dementia reported that only about 30% of participants, across 10 studies, were aware of hypertension as a risk factor [32]. The lack of awareness regarding many complications of hypertension, in particular, kidney disease and dementia, highlights the need for further education.

Midlife hypertension has been identified as playing a causal role in the development of dementia [33]. While public awareness of this link is low, an Australian survey found that 82% of respondents could recognize symptoms of dementia, surpassing recognition rates in six European countries, and 48% expressed concern about developing the condition [34]. This discrepancy, with low awareness of hypertension as a contributing factor alongside high recognition of dementia symptoms, highlights a critical gap in public understanding and presents an opportunity to engage the public on hypertension further.

Diagnosis and control of hypertension remain low both globally and in Australia [7,8]. The Lancet Commission on Hypertension recommends improving health literacy among the general public and states that “every adult should know their blood pressure” [35]. Our survey identified common misconceptions that should not be overlooked. Specifically, the silent nature of hypertension, with the usual lack of symptoms, highlights the importance of regular BP checks. However, Australia's May Measurement Month (2017–2019) report, based on data from 10 046 people, found that 33% had never previously had their BP checked, which is quite remarkable for a high-income country with an excellent health system [36]. Therefore, it remains highly relevant and essential to educate the public about hypertension as a silent killer, and engage, educate and raise awareness in more people through a global campaign like the May Measurement Month [37].

Furthermore, stress is not the main cause of hypertension; however, 41% of participants in our survey were unaware of this, and 24% incorrectly believed that it is. This common misconception may lead individuals to delay seeking treatment for high BP, assuming it will normalize once the stress subsides. Low awareness of the importance of medication adherence was also evident in our study, consistent with findings from other research [21]. Importantly, individuals already taking antihypertensive medications did not demonstrate better knowledge of hypertension than those who were not, highlighting missed opportunities for patient education within primary care. Given the well established issue of low medication adherence [38,39], enhancing awareness is a critical first step toward this gap [14,40]. The Australian National Hypertension Taskforce recommends an effective team-based care model that includes not only GPs but also nurses and pharmacists as part of the care team to improve patient follow-up, medication management, and medication adherence [11,41,42].

The knowledge gaps identified in our study should be addressed through patient education, awareness and health promotion campaigns. International initiatives, such as May Measurement Month, include education components that could be adapted to incorporate these specific gaps in understanding [37,43]. In Australia, the National Stroke Foundation “Australia's biggest blood pressure check”, launched in 2014, supports the global May Measurement Month initiative [44]. Previously, their “Know Your Numbers” campaign (2008–2010) successfully improved public knowledge of high BP risk factors; participants adopted healthier lifestyles, and more than 50% engaged in regular BP checks [45]. With the Taskforce's roadmap aiming for a 70% BP control rate by 2030, effectively addressing these identified knowledge gaps through public engagement initiatives may help achieve this goal [11].

Consistent with existing evidence, our study found that higher education and diagnosis of hypertension are positively associated with hypertension knowledge levels [20,21,23]. Our analysis did not find a statistically significant association between family history of cardiovascular-related diseases and hypertension knowledge, unlike previous research which indicated that a family history of hypertension enhances hypertension knowledge [20,21]. Furthermore, promoting a healthy lifestyle is important not only for the prevention and management of hypertension but also for overall health [46]. There remains a need to enhance public understanding of what constitutes a healthy diet. However, increasing dietary knowledge alone may be insufficient to improve the BP profile at the population level [47]. Future studies in this area will be required.

The strengths of our study include a sample of over 800 participants (albeit with a low response rate), and the use of a validated survey questionnaire alongside both machine-measured and self-reported data in all respondents. However, we recruited participants who self-measured their BP in a kiosk-based BP screening program, and the survey response rate was low, thus very likely introducing selection bias. Hence, hypertension knowledge may be overestimated and higher than that of the general population.

## CONCLUSION

Ongoing misconceptions persist among Australian adults regarding the risk factors and complications of hypertension, as well as the importance of medication adherence. There remains a need for health education in both primary care settings and the broader community to improve public knowledge, and future health promotion programs should specifically target the areas of poor understanding identified in our study to better engage the public and enhance health literacy. Target populations may include individuals with lower educational attainment and those who are unemployed. Addressing these knowledge gaps is essential to improving hypertension detection and management, and ultimately reducing the burden of cardiovascular disease in Australia.

## ACKNOWLEDGEMENTS

We would like to thank all research participants. We also extend our gratitude to Patrick Hannebery, Beth Kowalewski, and Dhanusha Battula at SiSU Health for their support in facilitating this research. Special thanks go to Linan Chen at The George Institute for Global Health for assistance with R programming.

Funding support: A.E.S. is funded by an Investigator Grant from the National Health and Medical Research Council of Australia (APP2017504). M.Z. is supported by Australian Government Research Training Program Scholarship.

Disclosures: A.E.S. has received speaker honoraria from Servier, Abbott, Sanofi, AstraZeneca, Medtronic, Omron, and Aktiia and serves on scientific advisory boards for Medtronic, AstraZeneca, SiSU Health and Sky Labs.

R.G.: This manuscript reflects the views of the author and does not represent the Department of Health, Disability and Ageing's views or policies.

### Conflicts of interest

There are no conflicts of interest.

## Supplementary Material

Supplemental Digital Content
