# Supplementary material for: Limited public understanding of the risk factors and complications of hypertension
Source: J Hypertens. 2026 Jan 9;44(3):512–9. doi: 10.1097/HJH.0000000000004233 (PMC12863606; doi:10.1097/HJH.0000000000004233)
Supplement: Supplemental Digital Content [file jhype-44-512-s001.docx]

**LIMITED PUBLIC UNDERSTANDING OF THE RISK FACTORS AND COMPLICATIONS OF HYPERTENSION**

Mingjuan Zeng^1,2^, Sonali R Gnanenthiran^1,2,3^, David K E Chan^4^, Ruth Griffiths^5^, Aletta E Schutte^1,2^

^1^The George Institute for Global Health, Sydney, NSW, Australia

^2^School of Population Health, University of New South Wales, Sydney, NSW, Australia

^3^Cardiology Department, Concord Repatriation General Hospital, NSW, Australia

^4^Stats Central, Mark Wainwright Analytical Centre, University of New South Wales, Sydney, NSW, Australia

^5^Department of Health, Disability and Ageing, Phillip, ACT, Australia

Corresponding author:

Professor AE Schutte, School of Population Health, High Street, Kensington Campus, University of New South Wales, Sydney 2052, NSW, Australia

Email: [a.schutte@unsw.edu.au](mailto:a.schutte@unsw.edu.au)

**SUPPLEMENTARY MATERIAL**

**Supplementary Table S1 SiSU Health Questionnaire**

1. Preferred name (What should we call you?)
2. Date of birth
3. Postcode
4. What was your sex recorded at birth?
   1. Male
   2. Female
5. How do you describe your gender?
   1. Man or Male
   2. Woman or Female
   3. Non-binary
   4. Prefer not to say
6. Are you pregnant? (Female users only)
7. When did you last see your doctor (GP)?
   1. Cannot remember
   2. 1 month ago
   3. 6 months ago
   4. 12 months ago
   5. More than 12 months ago
8. Are you currently taking medication for high blood pressure?
9. Have you had your blood pressure measured in the last 12 months?
10. Was your measurement in the high or at-risk range?
11. Do you have medications prescribed by your doctor?
12. Do you take medication exactly as it is described?
13. Do you stop taking it when you feel better?
14. Do you ever forget taking it?
15. Does medication make you feel weird?
16. Have you been diagnosed with Type 1 or Type 2 Diabetes?
17. Do you currently smoke cigarettes or any other tobacco products on a daily basis?
18. What is your descent?
    1. Aboriginal or Torres Strait Islander
    2. Pacific Islander or Maori
    3. None of the above
19. Have your parents, brothers or sisters been diagnosed with diabetes (Type 1 or Type 2)?
20. Where were you born?
    1. Australia
    2. Asia
    3. Middle East
    4. North Africa
    5. Southern Europe
    6. Other
21. Have you ever been found to have high blood glucose (sugar)? (e.g. in a health examination, during an illness, during pregnancy)
22. Do you eat vegetables or fruit every day?
23. On average, would you do at least 2.5 hours of physical activity a week? (e.g. 30 minutes a day on 5+ days a week)
24. What is your waist measurement?
    1. Less than 102 cm
    2. 102–110 cm
    3. More than 110 cm
25. Can high blood pressure cause any of the following? (Select all that apply)
    1. Heart Attacks
    2. Cancer
    3. Diabetes
    4. High cholesterol
    5. Allergy

**Supplementary Table S2 Survey questions and method for calculating individual knowledge scores**

| **Item questions** | **Answers** |
| --- | --- |
| 1. If your mother or father has high blood pressure, your chance of also having it is higher. | - True - False - I don’t know |
| 1. Young adults don’t get high blood pressure. | - True - False - I don’t know |
| 1. If you are overweight, you are 2 to 6 times more likely to develop high blood pressure. | - True - False - I don’t know |
| 1. Regular exercise can help reduce blood pressure. | - True - False - I don’t know |
| 1. Drinking alcohol lowers blood pressure. | - True - False - I don’t know |
| 1. High blood pressure is a man’s problem. | - True - False - I don’t know |
| 1. Stress is the main cause of high blood pressure. | - True - False - I don’t know |
| 1. Most people can tell when their blood pressure is high because they feel unwell. | - True - False - I don’t know |
| 1. High blood pressure is not life-threatening. | - True - False - I don’t know |
| 1. Increased blood pressure is the result of aging, so treatment is unnecessary. | - True - False - I don’t know |
| 1. If you take medication for high blood pressure, there is no need to change lifestyles. | - True - False - I don’t know |
| 1. People with high blood pressure do not need to take medicine if they exercise regularly. | - True - False - I don’t know |
| 1. A person is considered to have high blood pressure if either their systolic blood pressure is 140 or their diastolic is 90 or higher on two separate occasions. | - True - False - I don’t know |
| 1. #A person is diagnosed with high blood pressure if they have: | - a lot of headaches that persist over 6 months - family member with high blood pressure - constant stress and tension - high blood pressure measured at two different times at the doctor’s clinic - none of the above |
| 1. #A man reports that his blood pressure is 148 /78 mmHg when he checks it using the blood pressure machine in the pharmacy, 144 /66 mmHg in his family doctor’s office, and 132/ 74 mm Hg when he checks it at home. Which of the following statements is TRUE? | - It is common for blood pressure readings to vary like this - The highest blood pressure reading is the correct one - The lowest blood pressure reading is the correct one - He can be reassured that his blood pressure is normal - None of the above |
| 1. #High blood pressure can cause the following: (please tick all that apply) | - diabetes - gaining weight - damaging blood vessels - anxiety - stroke - dementia - none of the above |
| 1. #Which one of the following statements is TRUE about blood pressure medications? | - You should increase your own medication when your blood pressure is high. - You do not need to take medications on the days when your blood pressure readings are normal. - When you feel that you need to adjust your dose of medication, you first need to see your doctor. - Taking blood pressure medications for a long time can damage your body. - None of the above |
| 1. #Which of the following statements is FALSE about high blood pressure medications? | - Being on 2 or more types of blood pressure medication lower blood pressure more than only on 1. - Blood pressure medications need to be taken every day. - A person does not need to take blood pressure medications on the days they feel well. - Taking blood pressure medications together with a healthy lifestyle improves blood pressure. - None of the above |
| 1. #Which of the following diet changes DOES NOT lower your blood pressure? | - eat baked chicken instead of fried - stop eating potato chips - avoid adding table salt to food - eat fast food or fried foods - all of the above lower blood pressure |
| Each participant’s score was calculated based on the number of correct answers in the survey. For single-choice questions, one point was awarded for a correct answer and zero points for an incorrect or unknown answer. For the multiple-choice question on complications of high blood pressure, there were three correct answers and three incorrect answers. Correctly identifying a complication yielded one point, and not selecting an incorrect complication also yielded one point. Choosing ‘none of the above’ did not affect scoring. The minimum and maximum possible scores were 0 and 24, respectively. Participants who did not complete all the questions in the knowledge survey were excluded from the individual participant knowledge score analysis. | |

#questions completed by 798 participants, compared to 826 in the total study population.

**Supplementary Table S3 Comparison of the characteristics of respondents and non-respondents**

| **Variable (N=25,162)** | **Respondents n(%)** | **Non-respondents n(%)** | **P value** |
| --- | --- | --- | --- |
| **Total** | **826** | **24,336** |  |
| **Age group** |  |  |  |
| 18-34 years | 132(16.0) | 8219(33.8) | <0.0001 |
| 35-49 years | 218(26.4) | 8643(35.3) | <0.001 |
| 50-64 years | 264(32.0) | 5116(21.0) | <0.001 |
| 65 years and above | 212(25.7) | 2358(9.7) | <0.001 |
| **Female** | 408(49.4) | 9820(40.4) | <0.0001 |
| **Body composition^#^** |  |  |  |
| Body mass index, kg/m^2^ mean(SD) | 28.3(5.47) | 28.2(5.71) | 0.95 |
| **Blood pressure^#^**  Systolic BP, mmHg (mean) | 122 | 118 | <0.0001 |
| Diastolic BP, mmHg (mean) | 74 | 74 | 0.68 |
| BP ≥ 140/90 mmHg | 158(19.1) | 3380(13.9) | <0.0001 |
| **Use antihypertensive medications^#^** | 199(24.1) | 2830(11.6) | <0.0001 |
| **Geographical remoteness*^#^** |  |  |  |
| Major cities | 375(45.7) | 9321(38.3) | 0.34 |
| Inner regional | 334(40.7) | 11352(46.6) | 0.40 |
| Outer regional | 103(12.5) | 3171(13.0) | 0.74 |
| Remote and very remote communities | 9(1.0) | 180(0.7) | 0.35 |
| *Postcode was used to classify geographical remoteness according to the Australian Statistical Geography Standard – Remoteness Areas (ASGS-RA) framework.^30^  ^#^People who did not answer the question are included in the numerator; therefore, the total percentage does not sum to 100%. | | | |


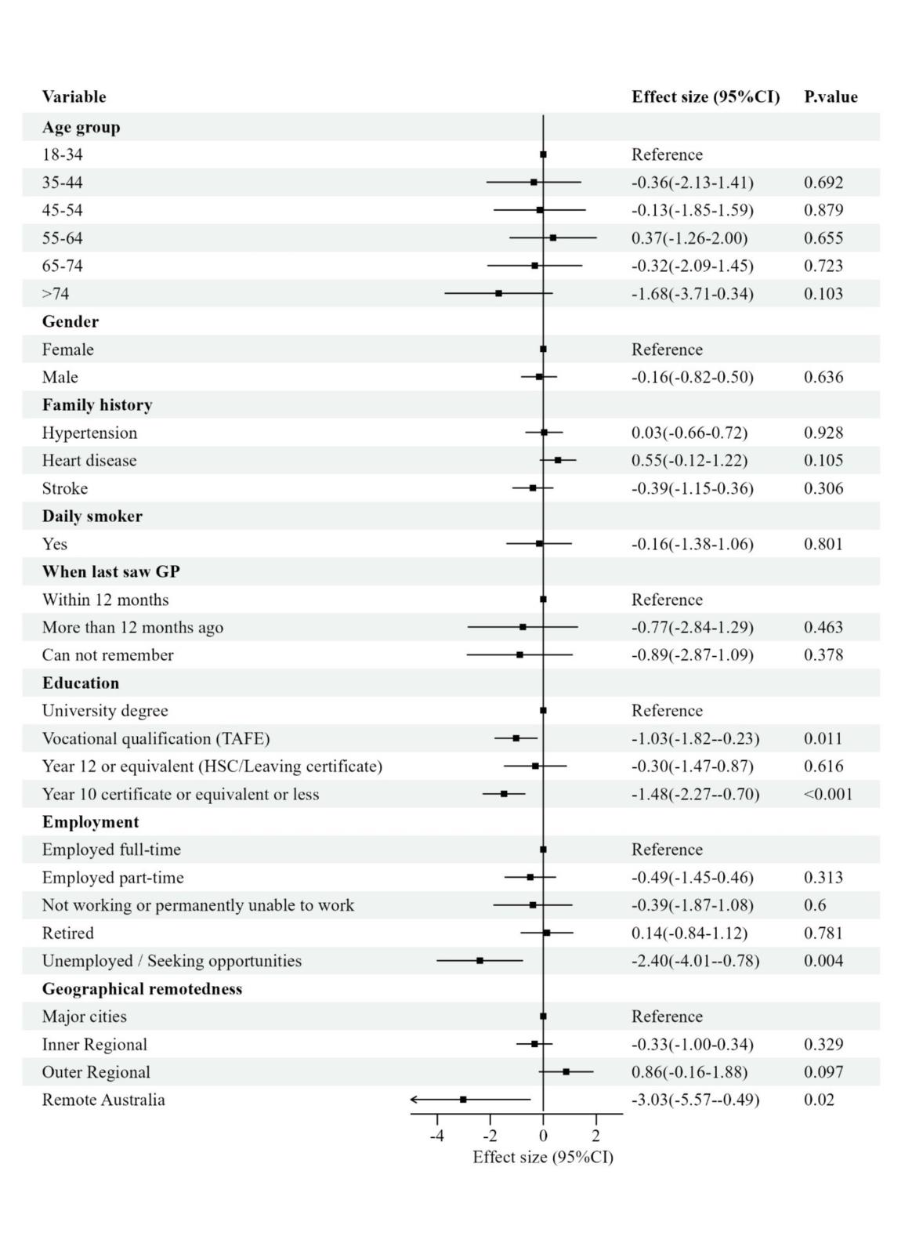


**Supplementary Figure S1 Effect sizes for predictors of hypertension knowledge scores among participants with a history of hypertension**


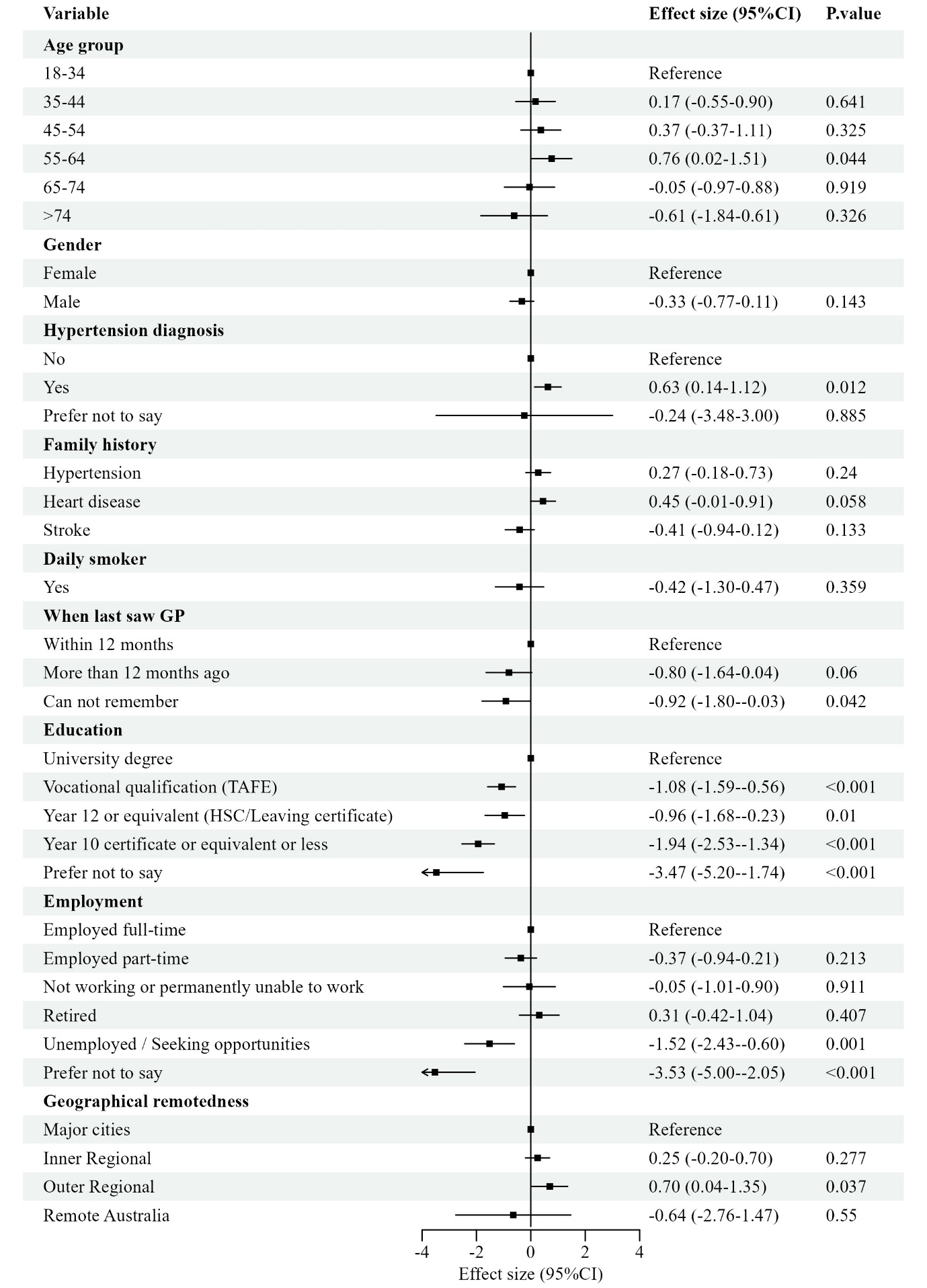


**Supplementary Figure S2 Effect sizes for predictors of hypertension knowledge scores: sensitivity analysis including participants who selected “Prefer not to say”**
